# Interactions between arbuscular mycorrhizal fungi and phosphate-soluble bacteria affect ginsenoside compositions by modulating the C:N:P stoichiometry in *Panax ginseng*

**DOI:** 10.3389/fmicb.2024.1426440

**Published:** 2024-10-02

**Authors:** Peng Mu, Guanzhong Ding, Yue Zhang, Qiao Jin, Zhengbo Liu, Yiming Guan, Linlin Zhang, Chijia Liang, Fan Zhou, Ning Liu

**Affiliations:** ^1^Laboratory of Medical Plant Cultivation, Institute of Special Animal and Plant Sciences, Chinese Academy of Agricultural Sciences, Changchun, China; ^2^State Key Laboratory of Black Soils Conservation and Utilization, Northeast Institute of Geography and Agroecology, Chinese Academy of Sciences, Changchun, China

**Keywords:** arbuscular mycorrhizal fungi, phosphate solubilizing bacteria, stoichiometry, ginsenoside, rhizosphere microbial community

## Abstract

The biomass production as well as the accumulation of secondary metabolites of plant is highly determined by the absorption of nutritional elements, in particular nitrogen (N) and phosphorus (P). Arbuscular mycorrhizal fungi (AMF) can absorb soluble P and transport it to plants, while phosphate solubilizing bacteria (PSB) can increase the content of solubilizing P in soil. Previous studies have identified the effects of either AMF or PSB inoculation on altering plant C:N:P stoichiometry, whether AMF interact with PSB in promoting plant growth and changing elemental concentration and composition of secondary metabolites by altering plant C:N:P stoichiometry remains ambiguous. In this study, we investigated the effects of inoculation of AMF, PSB, and their co-inoculation AMP (AMF and PSB) on the biomass growth, the C:N:P stoichiometry, the core microorganisms of rhizosphere soil, and the ginsenoside compositions of ginseng (*Panax ginseng*). The results showed that compared to control or single inoculation of AMF or PSB, co-inoculation of AMF and PSB significantly increased the AMF colonization rate on ginseng roots, increased the biomass of both above and under-ground parts of ginseng. Similarly, co-inoculation of AMF and PSB substantially increased the concentrations of N and P, reduced the ratios of C:P and N:P in the above-ground part of ginseng. The co-inoculation of AMF and PSB also increased concentrations of total ginsenosides and altered the compositions of ginsenosides in both the above and under-ground parts of ginseng. Analysis the rhizosphere microorganism showed that the co-inoculation of AMF and PSB recruited distinct core microorganisms that differ from the control and treatments with single inoculation of AMF or PSB. Our results suggested that PSB inoculation enhanced the positive effect of AMF in improving the absorption of nutrimental elements, altered the C:N:P stoichiometry and, ginsenosides concentration and composition of ginseng, influenced the plant rhizosphere microbial community. These findings offer valuable insights into enhancing plant biomass production and promoting secondary metabolites by improving the plant-fungi-bacterial relationships.

## 1 Introduction

The application of microorganism-based fertilizers is emerging as a nature-based and environment-friendly strategy for improving soil fertility and increasing crop yield and quality in sustainable agricultural systems (Khatoon et al., 2020). Plants and the rhizosphere microorganisms have a unique symbiotic relationship in which plants supply photosynthetically fixed carbon to microorganisms, while microorganisms promote plant growth by facilitating the acquisition of nutrients from soil (Zeng et al., 2022; Das et al., 2022). N and P are crucial biological elements, with levels in plant tissues strongly influencing growth, photosynthesis, and respiration rates. To enhance metabolic processes and growth rates, plants must synthesize a substantial quantity of P-rich RNA and ribosomes for constructing N-rich photosynthetic proteins. Therefore, the ratio of N and P elements serves as an indicator of plant growth rates (Elser et al., 2000; Xu et al., 2022). Beyond their role in synthesizing fundamental macromolecules, including nucleic acids and proteins, N and P are essential for most secondary metabolic activities in plants, such as the production of crucial compounds like alkaloids, terpenes, and flavonoids (Amtmann and Armengaud, 2009; Landi et al., 2019). While it’s well established that appropriate supplementation of N and P can enhance secondary metabolite content in medicinal plants (Sun et al., 2022; Xing et al., 2023), the specific influence of N:P stoichiometry on plant secondary metabolites remains unclear.

Arbuscular mycorrhizal fungi (AMF) are widely distributed plant root symbiotic fungi that form a common mycorrhizal network through extra-root hyphae, providing nutritional resources for host plants (Parniske, 2008; Zhou et al., 2022). However, AMF can only absorb soluble P and transport it to plants, thus its functions are primarily limited by soil soluble P content (Sharma et al., 2020). Releasing insoluble P in soil will likely promote the effects of AMF in promoting P uptake by plants. Phosphate solubilizing bacteria (PSB) account for more than 40% of soil-culturable bacteria (Kucey, 1983; Jorquera et al., 2008) and can convert soil organic and insoluble inorganic P by secreting metabolites such as organic acids, phosphatases, and phytases (Spohn et al., 2020). Recent studies have revealed positive relationships between PSB and AMF (Wahid et al., 2022), in which AMF hyphae stimulate the growth of PSB by releasing carbon-rich compounds; while PSB stimulates the germination of mycorrhizal spores, thus promoting the elongation of hyphae and increasing the mycorrhizal infection rate in plant roots (Zhang et al., 2016; Zhang et al., 2014; Sangwan and Prasanna, 2022). Thus, the co-application of AMF and PSB will likely promote P uptake by plants, resulting in a decrease in plant N:P stoichiometry.

Plant secondary metabolites are crucial for plant adaptation and defense. They also serve as significant sources of active ingredients in medicinal plants (Jamwal et al., 2018). It has been reported that both AMF and PSB can significantly affect C:N:P stoichiometry (Li et al., 2023; Yang et al., 2023). Notably, plant secondary metabolites are largely correlated with leaf stoichiometric traits (Rivas-Ubach et al., 2012). Prior investigations have indicated that AMF and PSB inoculation can enhance the accumulation of secondary metabolites in stevia (Tavarini et al., 2020) and aloe (Gupta et al., 2012). Thus, it is likely that the effects of AMF and PSB on plant secondary metabolites are mediated through changes in plant stoichiometry.

*Panax ginseng* Meyer is a perennial medicinal plant boasting an annual global market value of approximately $300 million (Bao et al., 2020). The medical value and the quality of *P. ginseng* are determined by the concentration and composition of a group of secondary metabolic molecules, i.e., ginsenoside (Shin et al., 2015). Ginsenosides comprise oleanane type and dammarane type (protopanaxadiol, PPD, and protopanaxatriol, PPT, respectively) (De Souza et al., 2011), and the ratio of PPD/PPT is a critical indicator for the medicinal quality of ginseng (Kim et al., 2018; Xu et al., 2017). However, cultivated ginseng is beset by continuous cropping obstacles and quality deterioration. Though previous research demonstrated that AMF biofertilizer application will improve ginseng growth (Liu et al., 2020), whether the quality, such as ginsenoside concentration and composition, will also be promoted is still unknown.

The objective of this study was to investigate the effects of inoculating AMF and PSB on the biomass growth, C:N:P stoichiometry and the ginsenoside content and compositions of ginseng. In this study, 16S rRNA sequencing technology and high-performance liquid chromatography were used to analyze the composition of soil bacteria and ginsenosides after inoculation with AMF and PSB. We hypothesized that: (1) co-inoculation of AMF and PSB will increase the AMF inoculation ratio on ginseng roots and alter composition and diversity of microorganism in rhizosphere soil; (2) co-inoculation of AMF and PSB will increase N and P uptake and change C:N:P stoichiometry of ginseng; (3) compared to single inoculation of AMF or PSB, co-inoculation of AMF and PSB will result in higher ginseng biomass and ginsenoside concentration as well as altered ginsenoside compositions. Our study delves deeper into the intricate mechanism through which AMF and PSB interact, which holds significant potential in boosting plant productivity and quality.

## 2 Materials and methods

### 2.1 Preparation of microbial agents

The phosphate-solubilizing bacteria used in the experiment were isolated from the rhizosphere of 10-year-old ginseng grown under the forest base (128°8′25″E, 43°7′45″N, 743.68 m) of Zixin Pharmaceutical Industry in Dunhua, Jilin Province. The phosphorus-solubilizing bacteria were identified as *Pseudomonas fluorescens* using molecular biology techniques. Following the modified method from previous studies (Świątczak et al., 2024). The bacteria were cultured in LB medium at 37°C and 200 rpm/min for 48 h and then centrifuged at 8,000 rpm/min for 10 min to discard the supernatant. The bacterial suspension was prepared using sterilized water and stored at 4°C. The bacterial suspension was adjusted to a final concentration of 10^8^ colony-forming units (CFU) per mL. The AMF biofertilizer (Agri, INOQ GmbH, Germany) was purchased from Germany and contained the arbuscular mycorrhizal fungi *Rhizoglomus irregular*, *Funneliformis mosseae*, and *F. caledonium* in vermiculite (diameter 1–2 mm) as the carrier material.

### 2.2 Pot experiments

Soil samples were collected from barren land (125.28° E, 43.71° N) at the Institute of Specialty Animal and Plant Sciences, Chinese Academy of Agricultural Sciences, which had not been previously planted. The soil basic physical and chemical properties were measured. The rhizosphere soil pH was 5.47, available N was 16.06 ± 0.34 mg kg^−1^, available P was 13.22 ± 1.78 mg kg^−1^, and available potassium 118.19 ± 6.24 mg kg^−1^. The experiment was conducted in 20 × 20 × 30 cm pots containing 1.5 kg of soil. Four treatments with four replicates each were established: (1) a sterile soil (Control), where 25 mL of sterile distilled water was added to each pot; (2) *Pseudomonas fluorescens* (PSB) treatment, where 25 mL of *Pseudomonas fluorescens* suspension was applied to each pot; (3) AMF bio-fertilizer (AMF) treatment, where 5 g of AMF bio-fertilizer was applied to each pot; and (4) a co-inoculated treatment with both *Pseudomonas fluorescens* and AMF bio-fertilizer (AMP), where 25 mL of *Pseudomonas fluorescens* bacterial suspension and 5 g of AMF biological fertilizer were added to each pot.

Two-year-old ginseng seedlings with uniform growth and no signs of disease were carefully selected for the experiment. Before planting, the shoot apexes of the seedlings were treated with 250 ppm gibberellin (GA) for 2–3 h to break their dormancy. The roots were then soaked in 75% anhydrous ethanol for 3 min, followed by washing with sterile distilled water, before planting in pots. Each pot was planted with five seedlings, and the pots were placed in the greenhouse, with a light intensity of 75.23 μmol/m^2^ s, temperature range of 23–25°C, and humidity of 70%. To avoid positional effects, the different treatments were randomly arranged, and their positions were changed weekly. The pots were watered with 200 mL per week.

### 2.3 Sampling of plant and rhizosphere soil samples

Ginseng was harvested at the red fruit stage, 90 days after planting, and both ginseng and rhizosphere soil samples were collected. The rhizosphere soil was collected using sterile containers and stored at −80°C for subsequent DNA extraction. The plant samples were divided into above and under-ground parts and cleaned with distilled water to remove surface contamination. After air drying, the dry weight, C, N, and P content, ginsenoside, and other indicators were measured.

### 2.4 Determination of ginseng growth index

AMF colonization rate was calculated according to the method described by Mcgonigle et al. (1990). The dry weight (DW) of both above-ground and under-ground ginseng samples was recorded, and after air-drying, they were crushed. The P content was extracted through digestion with mixed ultrapure acid (HNO_3_:HClO_4_, volume ratio 4:1), and then determined using the Mo-Sb colorimetric method on a UV–VIS spectrophotometer T9 (PUXI, China) (Bao, 2000). The C and N content was measured directly by an elemental analyzer (EURO EA 3000, Euro Vector, Italy) (Bontempo et al., 2023). The ginsenoside content was determined using high-performance liquid chromatography (Waters, Milford, Massachusetts, United States) (Sun et al., 2019). The ginsenosides monomers, including Rd., Rc, Rb1, Rb2, Rb3, Re, Rg1, Rg2, Rf were analyzed.

### 2.5 Illumina HiSeq sequencing

Following the protocol outlined by Gao et al. (2024), bacterial DNA was extracted from 0.5 g of rhizosphere soil stored at −80°C using the PowerSoil™ DNA extraction kit (MoBio Laboratories Inc., Carlsbad, CA, United States). The quantity and quality of the extracted DNA were determined using the NanoDrop ND-1000 UV–Vis spectrophotometer (NanoDrop Technologies, Wilmington, DE, United States). Polymerase chain reaction (PCR) was performed to amplify the V4 region of the 16S rRNA gene using specific bacterial primers 515F (GTGCCAGCMGCCGCGGTAA) and 806R (GGACTACHVGGGTWTCTAAT), each containing unique barcodes. The amplification reaction was carried out in a 30 μL reaction mixture consisting of 15 μL Phusion Master Mix (2x), 3 μL of each forward and reverse primer (2 μM final concentration), 10 μL of template DNA (10 ng), and 2 μL of nuclease-free water. The PCR protocol comprised an initial activation step at 98°C for 1 min, followed by 30 cycles of 98°C for 10 s, 50°C for 30 s, and 72°C for 30 s, and a final extension step at 72°C for 5 min. The resulting PCR product was purified from 2% agarose and gel generated using the GeneJET gel extraction kit (Thermo Fisher Scientific, Carlsbad, CA, USA). Sequence libraries were constructed using the TruSeq DNA PCR-free sample preparation kit (Illumina, Inc., San Diego, United States) (Dimitra et al., 2019). The quality of the library was assessed using the Qubit @ 2.0 fluorescence meter (Thermo Scientific) and the Agilent Bioanalyzer 2100 system, and sequencing was performed using the Illumina HiSeq 2500 platform, following the manufacturer instructions. The original readings of the 16S rRNA gene were deposited in the NCBI sequence read archive (SRA) database under the accession number PRJNA936811.

### 2.6 Sequencing data processing and bioinformatics analysis

The paired-end sequence readings were preprocessed using FLASH (version 1.2.7). Forward and reverse sequence readings with more than 10 base pairs overlapping and no base mismatches were merged into a single sequence. The sequencing data was processed using QIIME (version 1.8.0), and the UCHIME algorithm was used to remove low-quality and chimeric sequences. The sequences were sorted using the barcode and the high-quality sequence with the highest frequency in operational taxonomic unit (OTU) was selected as the representative sequence using Uparse (version 7.0.1001). The ribosomal database project (RDP) classifier algorithm was used to analyze the representative OTU sequences for the Silva (SSU128) 16S rRNA and UNITE (version 7.2) databases with a confidence threshold of 80%. This approach allowed for the determination of classification information and bacterial community composition at various levels, including phylum, class, order, family, genus, and species.

### 2.7 WGCNA analysis

To construct a co-expression network, we used the WGCNA package in R software to select the top 1,500 OTUs (excluding unidentified sequences) from the sequencing results of 16 ginseng rhizosphere soil microbiomes. A soft threshold power of *β* = 9 (*R*^2^ = 0.85) was chosen, and a hierarchical clustering tree was constructed using the dynamic hybrid cutting technique. We divided the co-expression network module and calculated the correlation between the module and different treatments. To screen core microorganisms, we used module membership (MM > 0.8), OTU significance (GS > 0.4), and OTU connectivity in the co-expression network.

### 2.8 Data analysis and statistics

Statistical analysis was conducted using SPSS v 20.0 (SPSS Inc., United States). Variance analyze and the Tukey comparison test were applied to analyze the data gathered from different treatments, with the resulting values presented as mean ± standard deviation (SD).

## 3 Results

### 3.1 AMF inoculation ratio, ginseng biomass and C:N:P stoichiometry

**Figure 1:**
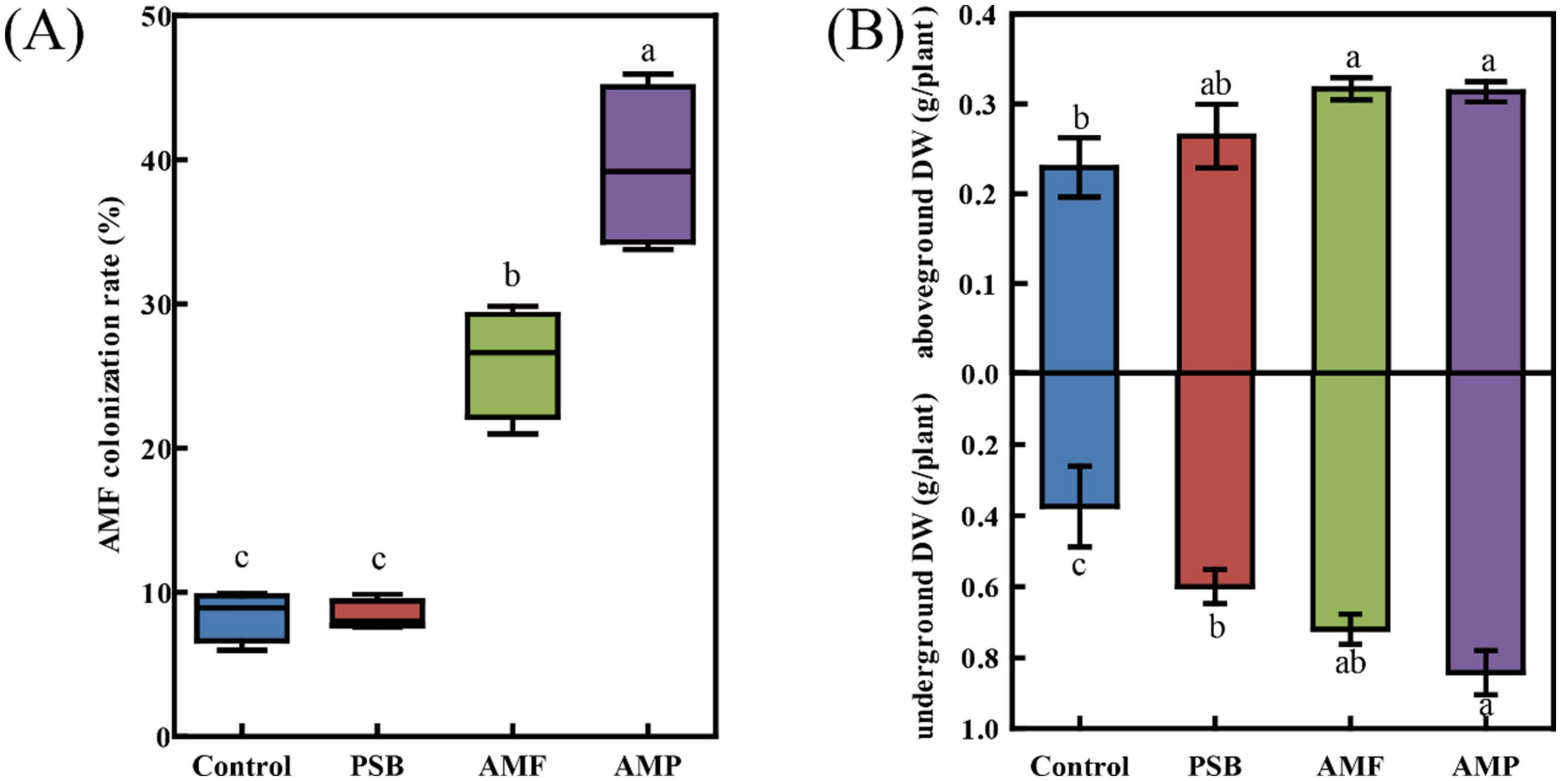
AMF gene colonization rate in ginseng root tissue **(A)** and Ginseng dry weight **(B)**. Data are means ± SD (*n* = 4) and different lowercase letters indicate the significance among treatments at *p* < 0.05. AMF, PSB, and AMP represent arbuscular mycorrhizal fungi, phosphate solubilizing bacteria, and the combination of AMF and PSB, respectively.

The AMF colonization rate of ginseng root was 8.4% in the control, and were 8.4, 26.0, and 39.5% in the PSB, AMF, and AMP treatments, respectively (Figure 1A). Compared to control, the PSB treatment increased dry weight of the under-ground part of ginseng by 60.0% (*p* < 0.05), but it had no significant effect on dry weight of the above-ground part. The AMF treatment increased the dry weight of both above-ground and under-ground parts by 38.3 and 92%, respectively; and the AMP treatment significantly increased the dry weight of the above-ground and under-ground parts by 36.8 and 125%, respectively (*p* < 0.05, Figure 1B).

**Figure 2:**
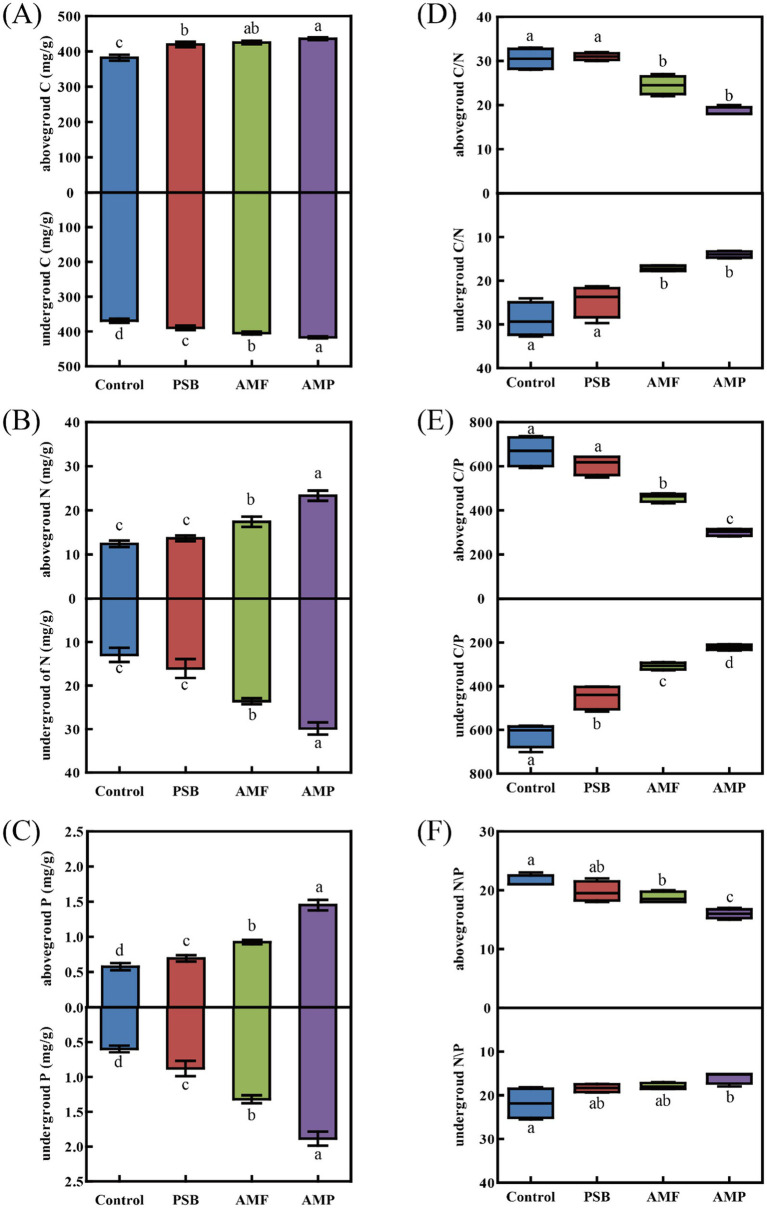
Difference in C **(A)**, N **(B)**, P **(C)**, C/N **(D)**, C/P **(E)**, and N/P **(F)** in above-ground and under-ground parts of ginseng in soil inoculated with AMF and/or PSB. Data are means ± SD (*n* = 4) and different lowercase letters indicate the significance among treatments at *p* < 0.05. AMF, PSB, and AMP represent arbuscular mycorrhizal fungi, phosphate solubilizing bacteria, and the combination of AMF and PSB, respectively.

Compared to control, the AMP treatment caused the most significantly increase of concentrations of C, N, and P of the above-ground part by 14.1, 87.8, and 151.8%, respectively; and those of the under-ground part by 12.8, 130.6, and 215.8%, respectively. Compared to control, the PSB treatment decreased the C:P ratio of the under-ground part by 27.6%. The AMF treatment significantly decreased C:N, C:P, and N:P ratios of the above-ground part by 21.0, 31.1, and 14.5%, respectively; and C:N and C:P ratios of the under-ground part by 43.1, and 55.6%, respectively (all *p* < 0.05, Figures 2D–F). The AMP treatment significantly decreased C:N, C:P, and N:P ratios of the above-ground part by 39.6, 48.2, and 16.0%, respectively; and those of the under-ground part by 53.0, 65.6, and 28.4%, respectively (all *p* < 0.05, Figures 2D–F).

### 3.2 Ginsenoside concentrations and the compositions

**Figure 3:**
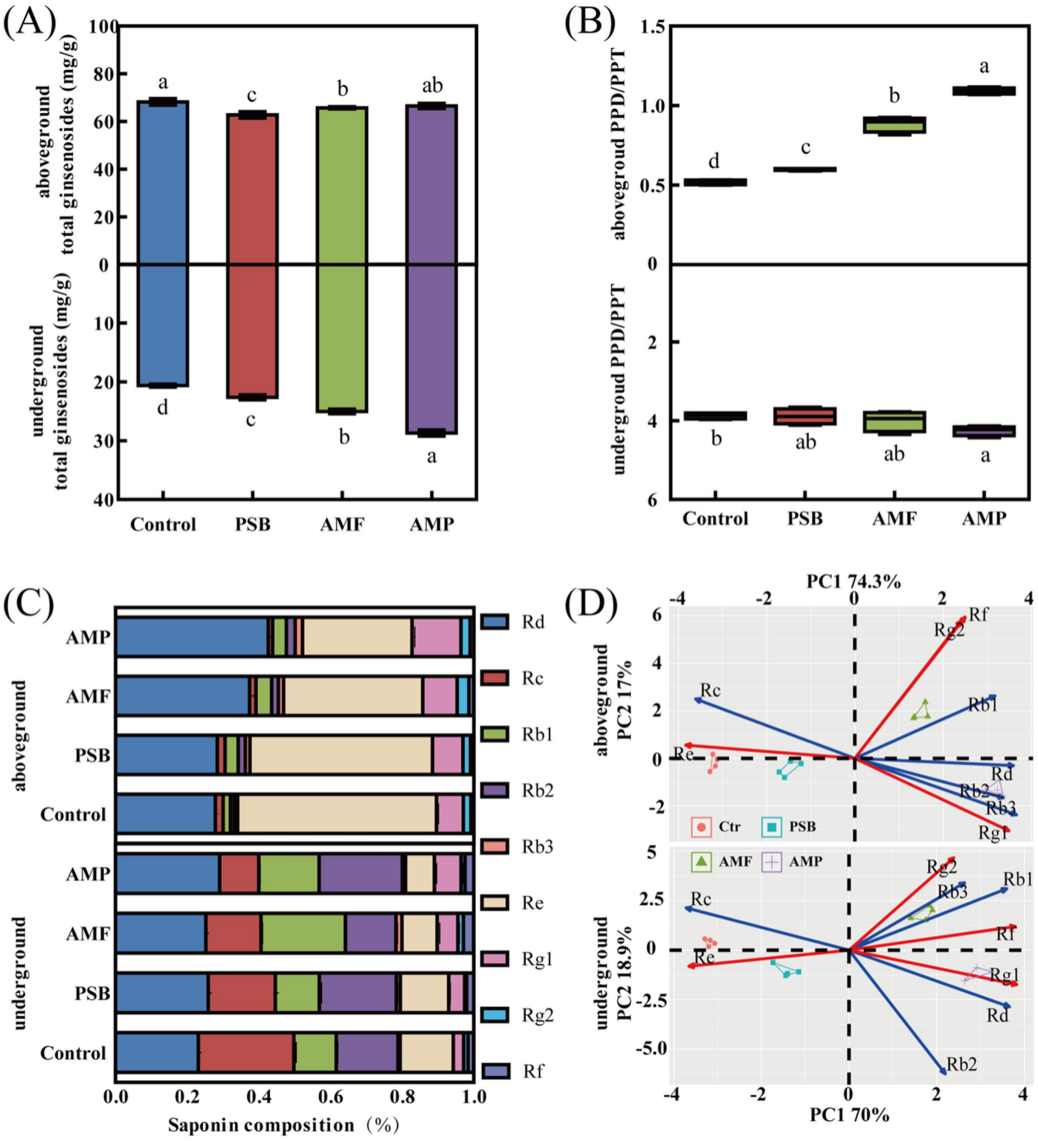
Differences in total ginsenoside **(A)**, PPD/PPT ratios **(B)**, Compositions of ginsenoside **(C)**, and PCA of ginsenoside **(D)** of aboveground and underground parts of ginseng in soil inoculated with AMF and/or PSB. Data are means ± SD (*n* = 4) and different lowercase letters indicate the significance among treatments at *p* < 0.05. Red and blue arrows indicated PPD, and PPT, respectively. AMF, PSB, and AMP represent arbuscular mycorrhizal fungi, phosphate solubilizing bacteria, and the combination of AMF and PSB, respectively.

Compared to control, the PSB, AMF, and AMP treatments significantly altered both the concentration of total ginsenoside and the PPD/PPT ratio (Figures 3A,B). Specifically, the PSB, AMF, and AMP treatments increased the concentration of total ginsenoside of the under-ground part by 9.7, 21.4, and 39.2%, respectively (*p* < 0.05, Figure 3A), but had little effect on the ginsenoside concentration of the above-ground part. Compared to control, PSB, AMF, and AMP treatments significantly increased the above-ground PPD/PPT ratio from 0.52 to 0.60, 0.88, and 1.09, respectively (*p* < 0.05, Figure 3B). However, the AMP treatment showing a slight effect (*p* < 0.05), the PSB and AMF showing no significant change on the under-ground PPD/PPT ratio (Figure 3B).

Compared to control, the PSB, AMF, and AMP treatments caused significant changes in the ginsenoside compositions (Figure 3C). The concentrations of Re and Rd. in the above-ground part were notably higher, constituting 27.8–42.6% and 30.6–55.5% of total ginsenosides, respectively. Compared to control, PSB, and AMF treatments, the AMP treatment significantly increased concentrations of Rd., Rb2, Rb3, and Rg1, while decreased concentrations of Rc and Re levels in above-ground parts (*p* < 0.05, Figure 3C; Supplementary Figure S1). The ginsenoside concentration was notably higher for Rd., Rc, Rb1, and Rb2 in the under-ground part, accounting for 23.1–29.16%, 10.9–26.7, 11.9–23.5%, and 14.1–23.2% of total ginsenosides, respectively. The AMP treatment significantly enhanced the concentration of Rd., Rb2, and Rg1, while simultaneously reducing the concentration of Rc in the under-ground part compared to the control, PSB, and AMF treatments (*p* < 0.05, Figure 3C; Supplementary Figure S1).

The PCA on the nine ginsenoside concentrations revealed that the four treatments in above-ground and under-ground parts were distinguished along the first two PC axes (Figure 3D). In the above-ground part, the first two principal components (PC1 and PC2) explained 89% of the total variation, PC1 having the highest loadings on Rb3 and Rd., and PC2 having the highest loadings on Rf and Rg2 (Supplementary Table S1). In the under-ground part, PC1 and PC2 explained 91% of the total variation, PC1 having the highest loadings on Rg1 and Rf, PC2 having the highest loadings on Rg2 and Rb3 (Supplementary Table S1).

### 3.3 The rhizosphere core microorganisms

**Figure 4:**
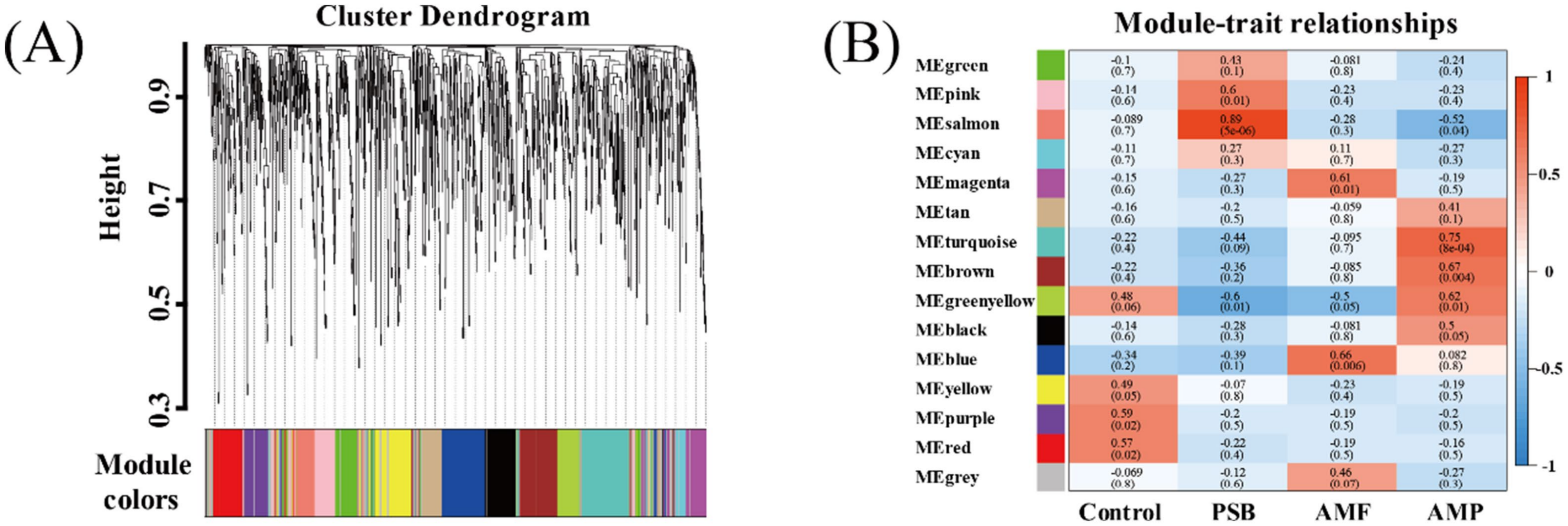
Key module identification based on WGCNA. Module partitioning **(A)** and module correlation to processing **(B)**. AMF, PSB, and AMP represent arbuscular mycorrhizal fungi, phosphate solubilizing bacteria, and the combination of AMF and PSB, respectively.

Compared to control, the AMF, PSB, and AMP treatments decreased the diversity of ginseng rhizosphere microorganisms and changed in the structure and composition of microbial communities (Supplementary Figure S2). Used WGCNA co-expression network analysis to generate 15 modules (Figure 4A). A significant correlation between the four treatments and different bacterial modules was observed. Specifically, different treatments were significantly correlated with different bacterial modules, and the purple module (79 OTUs) was highly correlated with control (*r* = 0.59); the salmon module composed of 72 OTUs exhibited a higher correlation with the PSB treatment (*r* = 0.89); the blue module consisted of 152 OTUs was strongly associated with the AMF treatment (*r* = 0.59); the turquoise modules encompassed 177 OTUs demonstrated substantial correlation with the AMP treatment (*r* = 0.75).

**Figure 5:**
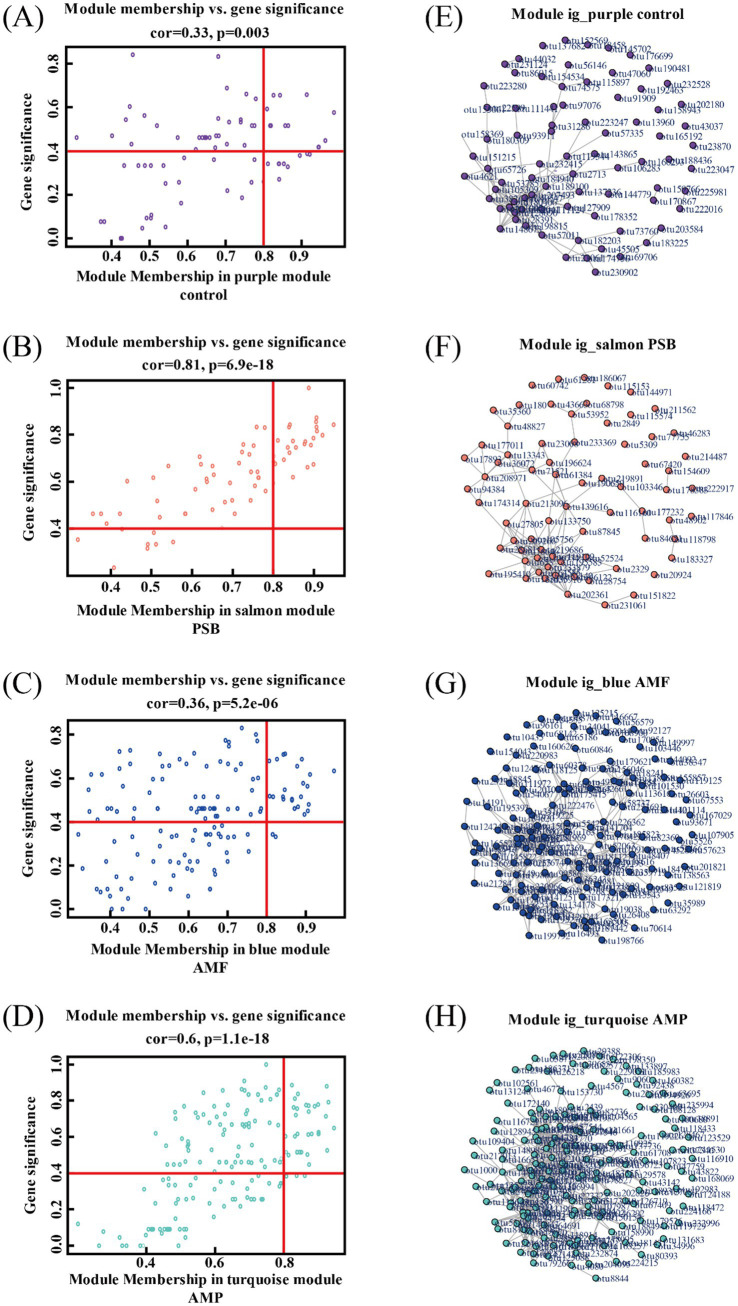
Module members and OTU saliency of individual OTUs in modules purple **(A)**, salmon **(B)**, blue **(C)**, and turquoise **(D)**. Visualization of fully weighted networks for modules purple **(E)**, salmon **(F)**, blue **(G)**, and turquoise **(H)**. AMF, PSB, and AMP represent arbuscular mycorrhizal fungi, phosphate solubilizing bacteria, and the combination of AMF and PSB, respectively.

The analysis revealed 26, 24, and 25 candidate core bacterial OTUs of AMF, PSB, and AMP, respectively, exceeding the count observed in control with 14 core bacterial OTUs (Figures 5A–D). Further, identified different core bacterial genera among the candidate core OTUs from control, PSB, AMF, and AMP treatments, and selected the top 5 OTUs as candidate core OTUs (Figures 5E–H). The core microorganisms were identified according to the above two methods. The core microorganisms in the control were *Jahnella thaxteri* and *Labilithrix Sorangiineae* combined with species annotation. The core microorganisms in the PSB was *Pseudomonas bacterium* ASP38; The core microorganisms in the AMF were *Spirochaeta lutea*, *Pseudomonas boreopolis*, and *Acidibacter* sp.; The core microorganisms in the AMP were *Acidobacteria bacterium* WY67, *Rhizobium Sinorhizobium* XLL-7, and *Paeniglutamicibacter bacterium* d8829 (Table 1). Spearman analysis showed a strong correlation between the core microorganisms under different treatments and the stoichiometric ratio of ginseng and ginsenoside concentration (Supplementary Figure S3).

**Table 1:** Core microorganisms corresponding to different treatments.

Treatment	OTUs	Degree	Kingdom	Phylum	Class	Order	Family	Genus	Species
Control	otu 207493	17	Bacteria	Proteobacteria	Deltaproteobacteria	Myxococcales	Polyangiaceae	*Jahnella*	*Jahnella thaxteri*
	otu 62041	16	Bacteria	Proteobacteria	Deltaproteobacteria	Myxococcales	Polyangiaceae	*Labilithrix*	*Labilithrix Sorangiineae*
	otu 128090	16	Bacteria	Proteobacteria	Deltaproteobacteria	Myxococcales	Polyangiaceae	*Jahnella*	*Jahnella thaxteri*
PSB	otu 119109	18	Bacteria	Proteobacteria	Gammaproteobacteria	Pseudomonadales	Pseudomonadaceae	*Pseudomonas*	*bacterium* ASP38
	otu 219686	17	Bacteria	Proteobacteria	Gammaproteobacteria	Pseudomonadales	Pseudomonadaceae	*Pseudomonas*	*bacterium* ASP38
	otu 195583	16	Bacteria	Proteobacteria	Gammaproteobacteria	Pseudomonadales	Pseudomonadaceae	*Pseudomonas*	*bacterium* ASP38
AMF	otu 19800	22	Bacteria	Spirochaetae	Spirochaetes	Spirochaetales	Spirochaetaceae	*Salinispira*	*Spirochaeta lutea*
	otu 143152	22	Bacteria	Proteobacteria	Gammaproteobacteria	Xanthomonadales	Xanthomonadaceae	*Xylella*	*Pseudomonas boreopolis*
	otu 174027	20	Bacteria	Proteobacteria	Gammaproteobacteria	Xanthomonadales	Xanthomonadaceae	*Acidibacter*	*Acidibacter* sp.
AMP	otu 210467	26	Bacteria	Acidobacteria	Holophagae	Subgroup	NA	*Acidobacteria*	*bacterium* WY67
	otu 59471	23	Bacteria	Proteobacteria	Alphaproteobacteria	Rhizobiales	Rhizobiaceae	*Rhizobium*	*Sinorhizobium* XLL-7
	otu 86918	23	Bacteria	Actinobacteria	Actinobacteria	Micrococcales	Micrococcaceae	*Paeniglutamicibacter*	*bacterium* d8829

AMF, PSB, and AMP represent arbuscular mycorrhizal fungi, phosphate solubilizing bacteria, and the combination of AMF and PSB, respectively.

## 4 Discussion

### 4.1 The N: P stoichiometry was decreased with co-inoculation AMF and PSB

There is considerable evidence supporting the growth-promoting mechanism of AMF and PSB on plants (Rawat et al., 2021; Mitra et al., 2021). Our study found that co-inoculation of AMF and PSB promoted AMF colonization rate on ginseng roots (Figure 1), indicating a synergistic interaction effect between AMF and PSB, which has been reported in previous studies (Zhang et al., 2016). In our study, AMF alone or co-inoculation with PSB both significantly increased the dry weight of the under-ground part of ginseng likely resulted from the increased N and P concentrations in both the above and under-ground parts of ginseng (Figure 2). Similarly, Nacoon et al. (2021) also showed that AMF and PSB co-inoculation promote growth and nutrients uptake of *Helianthus tuberosus* in fields. This interaction may be explained by the fact that AMF secreted carbon derived from plant photosynthesis into the soil, providing nutrient substrates for PSB to dissolve insoluble P continuously (Wang et al., 2016). The PSB, in turn, increased mycorrhizal colonization and spore density, facilitating better N and P transport from soil to plants (Nacoon et al., 2021).

Changes in the elemental concentration of plants naturally affect the C:N:P ratios. Higher C:N and C:P ratios indicate a greater utilization of N and P by the plant (Ma et al., 2019). The N:P ratio can help determine whether plant growth is limited by N or P and also provide insight into the plant growth rate (Gusewell, 2004). In the present study, the co-inoculation of AMF and PSB significantly increased the concentrations of N and P of ginseng, resulting in decreased C:N and C:P ratios, which is consistent with the outcomes of former studies (Rabaa et al., 2019). In our study, the N:P ratio of the above-ground part of the control group was 22, higher than the threshold ratio of 16 or 20 which was suggested to be P limitation by plants (Koerselman and Meuleman, 1996; Gusewell, 2004), suggesting that the growth of ginseng was P limited. While AMP treatment reduced the N:P ratio to 16 (Figure 2F), signifying that AMF and PSB co-inoculation significantly alleviate the P limitation of ginseng, which promoted ginseng growth.

### 4.2 Ginsenosides concentration was increased and composition was changed with co-inoculation AMF and PSB

The highest ginsenoside concentration in the under-ground parts of ginseng in the AMP treatment suggested that AMF likely interacts with PSB in promote the synthesis of secondary metabolites in ginseng (Figure 3). These results are in line with previous findings that AMF and PSB co-inoculation significantly increased the contents of phenols and vitamin C in eggplants (Sharma et al., 2022), and the contents of thymol and *γ*-terpinene in fennel (Rezaei-Chiyaneh et al., 2021). In this study, the strong increase in total ginsenoside concentration may result from the higher concentrations of N and P in the AMP treatment. It has been shown that concentrations of both N and P play pivotal roles in modulating key enzyme activity involved in the ginsenoside synthesis pathway, impacting ginsenoside synthesis and accumulation in ginseng plant (Han et al., 2010; Tansakul et al., 2006). N deficiency inhibits ginseng growth and photosynthetic capacity, leading to reduced plant photosynthetic product synthesis and diminished ginsenoside production (Li et al., 2020). In *Panax notoginseng*, ginsenoside synthesis is notably correlated with soil P levels (Zhong and Zhu, 1995).

More importantly, the present study also showed that the inoculations of AMF and PSB also altered the ratio of ginsenosides PPD and PPT (Figure 3). Kochan et al. (2016) discovered that the content of ginsenoside Re in ginseng hairy roots decreased, and the content of ginsenoside Rd. increased with an increase in the proportion of P in the medium. Kolodziej et al. (2015) reported that the use of P fertilizer elevated the PPD/PPT ratio. The C:N:P ratios changed by AMF inoculation and nutrient availability to induce secondary metabolite production were also reported (Fritz et al., 2006; Wei et al., 2019). Thus, the altered ratios of PPD/PPT found in present study may well be explained by the reduced N:P. Previous studies further evidenced that N concentration could regulate cytochrome P450 enzyme which involved biosynthesis ginsenoside (Kochan et al., 2016), and that PPD-type saponins are can be subsequently converted into PPT-type saponins under certain N or P concentration levels (Rahimi et al., 2015; Sun et al., 2024). We thus speculate that the changed C:N:P ratios are responsible for transformations of PPD to PPT saponins in ginseng, however, further researches are needed to investigate the detailed mechanisms in but not limited to ginseng.

### 4.3 AMF and PSB recruit N and P associated core microbes

The changed C:N:P stoichiometry and ginsenoside compositions may also attributed to the significant changes of rhizosphere microorganisms. In the present study, the inoculation of AMF and PSB were found significantly changing the composition of rhizosphere microbial communities (Supplementary Figure S2). Song et al. (2021) observed that introducing PSB (*Klebsiella* sp.) increased the abundance of actinomycetes in the rhizosphere of elm. Xue et al. (2023) discovered that AMF significantly increased the presence of *Rhodobacter*, *Archangium*, and *Longimicrobium* in the rhizosphere of *Astragalus*. Zhang et al. (2016) found that C sources, such as galactose, glucose, and trehalose, released by *Rhizophagus intraradices* hyphae, may induce changes in specific rhizosphere bacterial communities. In this study, we found that PSB and AMF facilitated the recruitment of a bacterium species *Pseudomonas* spp. which is a phosphate-solubilizing microorganism (Korshunova et al., 2021), that able to dissolve insoluble P in soil by secreting organic acids (Jiang et al., 2021). Moreover, *Pseudomonas* can secrete phosphatase to mineralize organic P in soil, improving the utilization efficiency of P in soil (Rawat et al., 2021). Additionally, Liu et al. (2024) found that inoculating *Pseudomonas thivervalensis* increased the total saponin content in ginseng. *Rhizobium Sinorhizobium* is a unique core microorganism of AMF and PSB co-inoculation. *Rhizobium Sinorhizobium* is a nitrogen-fixing bacterium that can reproduce in plant cells, whereas bacterial cells can fix nitrogen in the atmosphere into ammonium, which plants can use to enhance their absorption capacity for N (Xue and Biondi, 2019). In together, these suggest that the changed rhizosphere microorganisms are benefiting N and P absorption by ginseng.

Core microorganisms may not have direct functional roles but can influence soil properties and plant growth by either stimulating or inhibiting other functional species (Hassani et al., 2018). In this study, Proteobacteria and Actinobacteria were the dominant bacterial phyla in the ginseng rhizosphere (Supplementary Figure S2). Our analysis revealed that the core microbiota across the four treatments were closely associated with these dominant phyla, forming an interconnected microbial community (Figure 5). Furthermore, the three core microorganisms under AMF and PSB co-inoculation were significantly correlated with *Bacillus* spp. and *Rhizobium* spp. (Figure 5F). *Bacillus megaterium* (Jiang et al., 2019) and *Bacillus amyloliquefaciens* (Luo et al., 2022). *Bacillus* spp. promote the mineralization of soil organic P and release phosphate. *Rhizobium* spp. can produce carboxylic acids release them into the soil, and release phosphate in iron, aluminum, and calcium complexes (Guimaraes et al., 2022). The above results indicate that AMF and PSB induce changes in microbial communities by recruiting core microorganisms associated with N and P cycling, promotes the absorption of N and P, change the C:N:P stoichiometry of the plant, and affects its ginsenoside composition. Our findings not only provide clues for the use of adequate microbial inoculation could recruit the core microorganisms that promote plant growth and enhance nutrients uptake, but also help to understand underlying mechanisms driving the association between plant stoichiometry and secondary metabolite in terrestrial ecosystems.

## 5 Conclusion

In general, the presenet study provide strong evidence that co-inoculation of AMF and PSB, is more effective than single inoculation of AMF or PSB in promoting the growth and ginsenoside accumulation of ginseng. Specifically, co-inoculation of AMF and PSB, significantly enhanced the absorption of N and P nutrients, leading significant decreases in C:N, C:P, and N:P ratios, and regulated the core microorganisms of the ginseng rhizosphere soil. On the other hand, co-inoculation of AMF and PSB also promoted total ginsenoside concentrations, changed the composition of ginsenosides, and increased the ratio of PPD-type and PPT-type ginsenoside. Our study further elucidates the mechanism by which AMF and PSB interact with each other to impact plant growth and secondary metabolism and provides important evidence for the synthesis and assembly of the core microbiome to enhance plant productivity and quality.

## Data Availability

The datasets presented in this study can be found in online repositories. The names of the repository/repositories and accession number(s) can be found below: https://www.ncbi.nlm.nih.gov/, PRJNA936811.
