# Supplementary material for: Interactions between arbuscular mycorrhizal fungi and phosphate-soluble bacteria affect ginsenoside compositions by modulating the C:N:P stoichiometry in Panax ginseng
Source: Front Microbiol. 2024 Oct 2;15:1426440. doi: 10.3389/fmicb.2024.1426440 (PMC11479886; doi:10.3389/fmicb.2024.1426440)
Supplement: Supplementary file 1 [file Data_Sheet_1.docx]

**Supplementary Material**

**Table S1.** Loading matrix of the principal components for the content of main ginsenosides in the above-ground and under-ground parts of ginseng.

| group | aboveground | | | underground | | |
| --- | --- | --- | --- | --- | --- | --- |
|  | PC1 | PC2 | PC3 | PC1 | PC2 | PC3 |
| Rb1 | 0.3181925 | 0.26301891 | -0.56503644 | 0.3596562 | 0.31052759 | -0.04144647 |
| Rb2 | 0.3372732 | -0.16559838 | -0.54881047 | 0.2195549 | -0.62510591 | 0.06519151 |
| Rb3 | 0.3668375 | -0.23736641 | 0.08405013 | 0.263464 | 0.34072705 | -0.71686857 |
| Rc | -0.3611697 | 0.24989589 | -0.10356413 | -0.3717917 | 0.21371221 | 0.1418233 |
| Rd | 0.3599231 | -0.03041414 | 0.42626919 | 0.3658788 | -0.28622803 | 0.05344305 |
| Re | -0.3835959 | 0.05557664 | 0.05051217 | -0.3653173 | -0.08144158 | -0.26981384 |
| Rf | 0.2504904 | 0.59463826 | -0.01997659 | 0.3793739 | 0.11947418 | -0.11064925 |
| Rg1 | 0.3498242 | -0.30475844 | 0.24151449 | 0.3827713 | -0.17398926 | 0.05472354 |
| Rg2 | 0.2411931 | 0.57803227 | 0.34462403 | 0.2388268 | 0.46684239 | 0.60755598 |


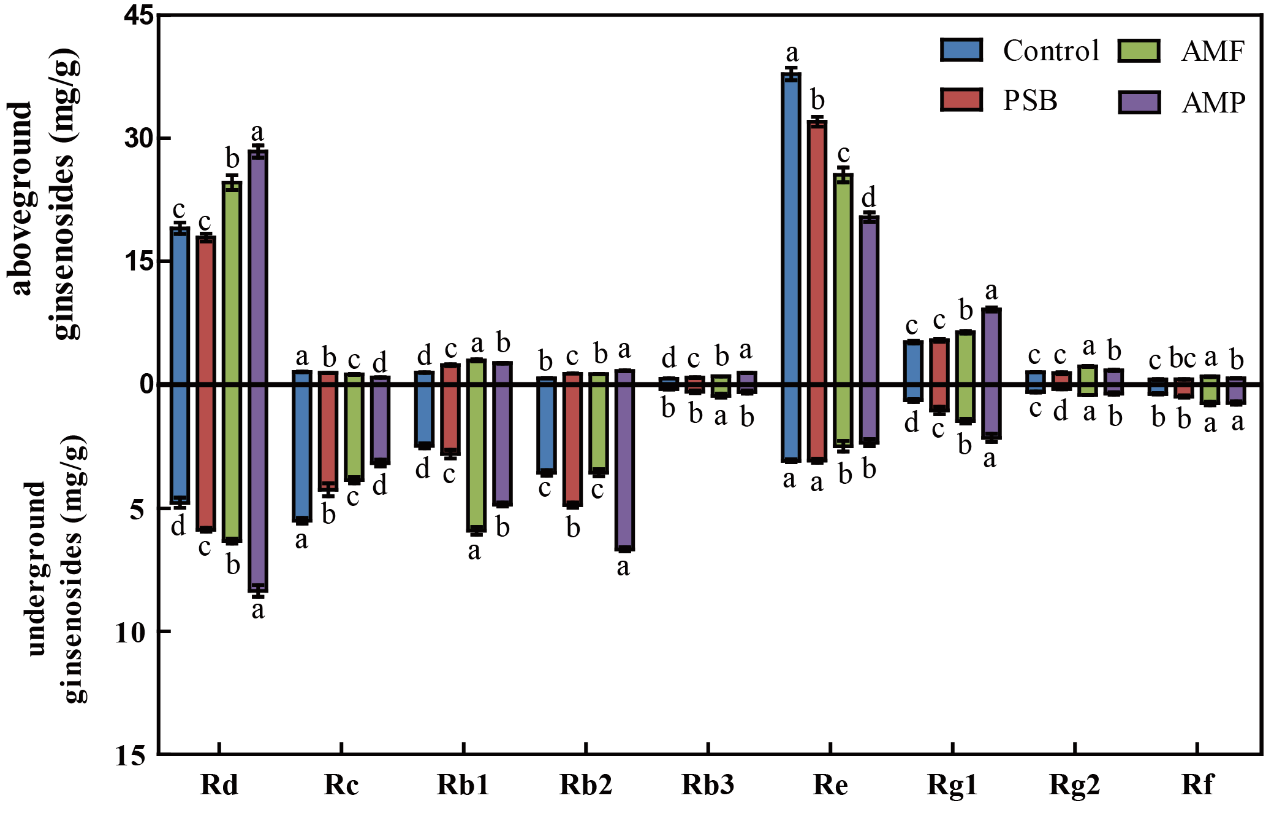


**Fig. S1** Differences in monomeric ginsenoside Content of above-ground and under-ground parts of ginseng in soil inoculated with AMF and/or PSB. Data are means ± SD (n = 4) and different lowercase letters indicate the significance among treatments at *P*< 0.05. AMF, PSB, and AMP represent Arbuscular mycorrhizal fungi, phosphate solubilizing bacteria, and the combination of AMF and PSB, respectively.


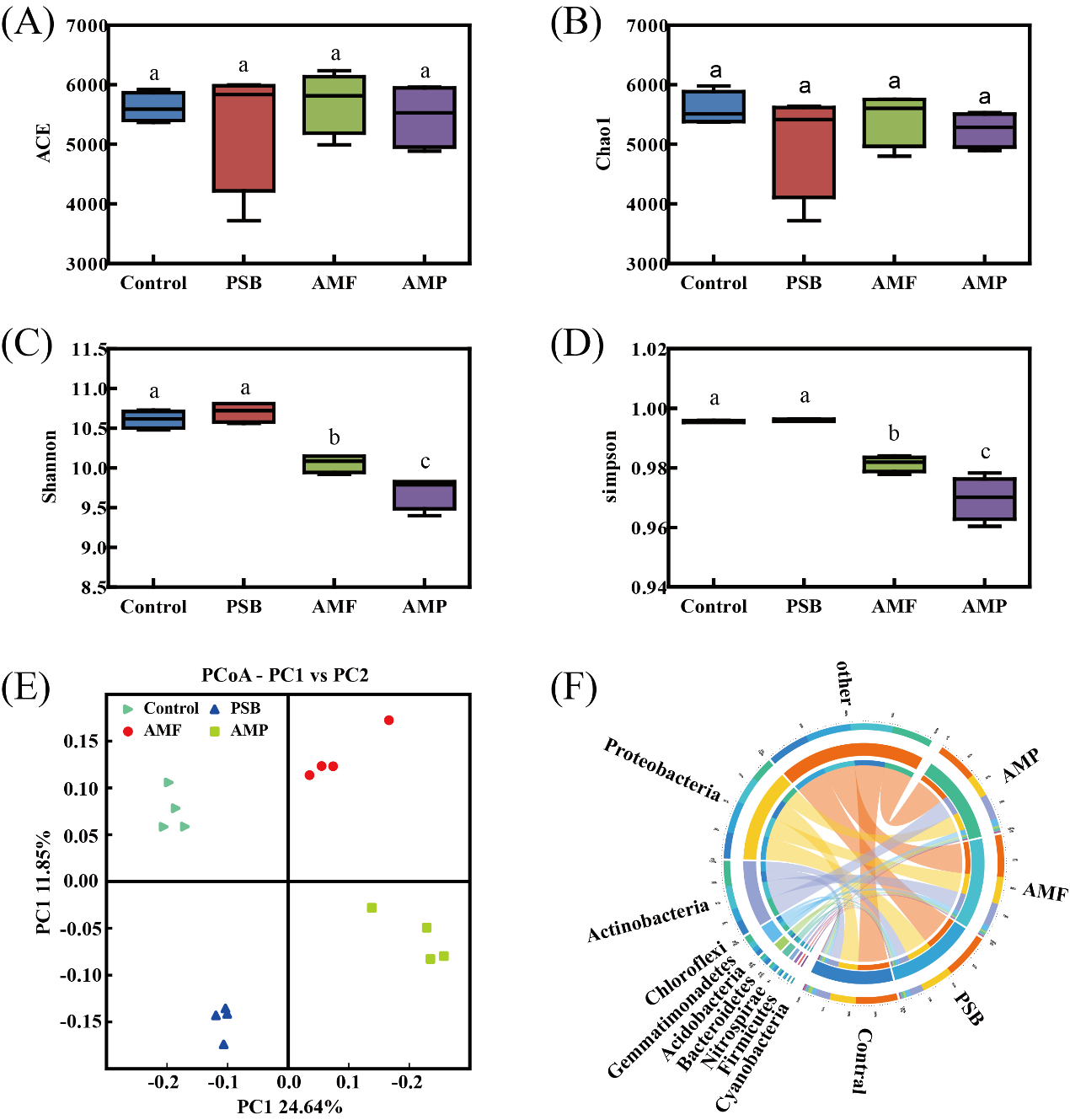


**Fig. S2** Differences in ACE **(A)**, Chao1 **(B)**, Shannon **(C)**, Simpson **(D)**, PCoA **(E)**, and compositions **(F)** of rhizosphere microorganisms in ginseng inoculated soil with AMF and/or PSB. Data are means ± SD (n = 4) and different lowercase letters indicate the significance among treatments at *P*< 0.05. AMF, PSB, and AMP represent Arbuscular mycorrhizal fungi, phosphate solubilizing bacteria, and the combination of AMF and PSB, respectively.


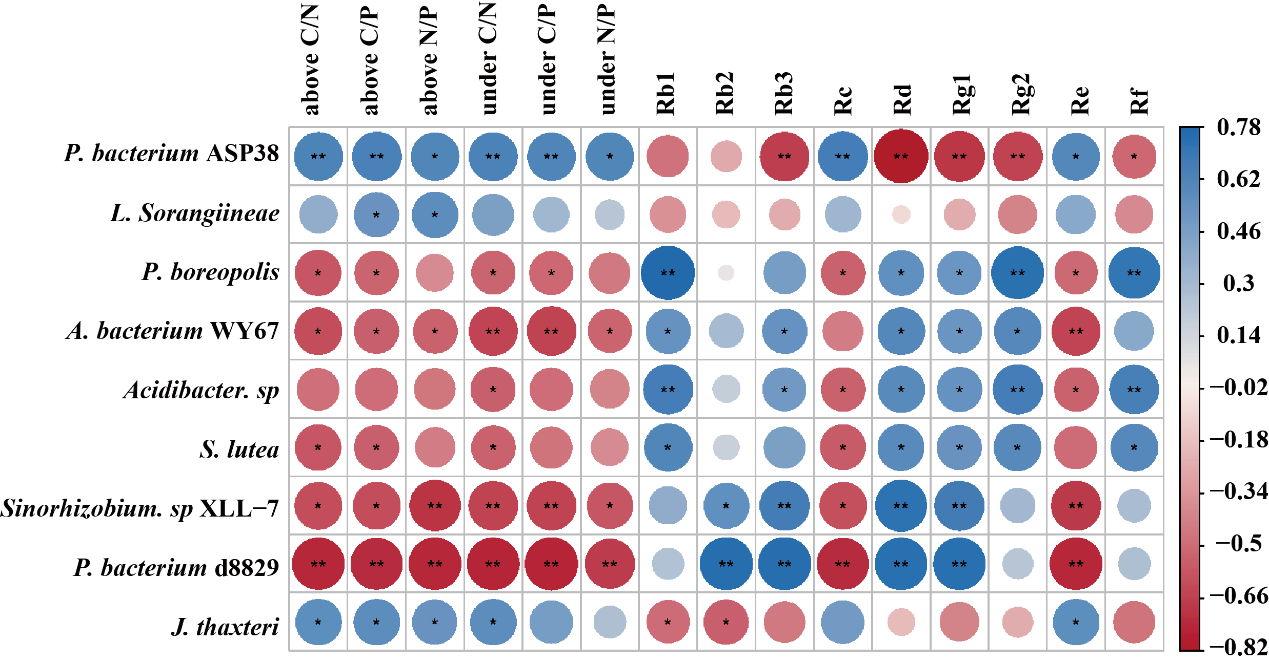


**Fig. S3** Correlation analysis of C:N, C:P, and N:P with different monomer ginsenoside in ginseng's above-ground and under-ground parts**.**
